# The Operative Treatment of Corneal Astigmatism

**Published:** 1901-06-08

**Authors:** 


Lancet, June

### THE OPERATIVE TREATMENT OF CORNEAL ASTIGMATISM.

It is well known that the greater part of astigmatism is, under normal conditions, due to faulty curvature of the cornea. It is also well known that astigmatism is often produced by operations involving the cornea, and that a considerable experience has thus been accumulated in regard to the effects on corneal curvature, and, therefore, on refraction which may be expected to result from incisions penetrating the cornea in various directions. Hence not unnaturally various operators have endeavoured to cure astigmatism by appropriately placed corneal incisions, made usually at one or both ends of the meridian of stronger refraction, and in some cases the results have been good, the astigmatism becoming less or even disappearing altogether. Whether it is justifiable as the result of such experience to make a perforating corneal incision for the cure of astigmatism is, however, a matter on which there is likely to be some difference of opinion. Dr. A. Breuer[1] holds that it is not, and he urges in its place the use of the galvano-cautery at a dull red heat. He employs the ordinary loop of fine platinum wire, presenting at its tip a surface of about one millimetre, and makes a small punctiform burn in the limbus or in the cornea, penetrating to about one half of the corneal thickness. The cauterisation wound invariably healed in from one to two days, and about three weeks after the operation the scar could be detected only by a very close inspection or by focal illumination. The best results were obtained in cases of compound hypermetropic astigmatism. In estimating the desirability of the operation in any given case it may be well to bear in mind that it is always necessary to produce a considerable over-effect, as a considerable part of the first result disappears in time, the proportion which is thus lost seeming to be greater in children than in adults. Further, the more superficial and peripheral the cauterisation the more likely is it that the result will disappear in time. If, however, the cauterisation extend through about one-half of the corneal thickness a permanent effect will be obtained. Evidently, as in all operations in which an over-effect has to be aimed at, the due estimation of the amount of immediate effect required is a matter demanding considerable judgment.

[1] Lancet, June 1.

